# Supplementary material for: Collaborative intelligence in AI: Evaluating the performance of a council of AIs on the USMLE
Source: PLOS Digit Health. 2025 Oct 9;4(10):e0000787. doi: 10.1371/journal.pdig.0000787 (PMC12510544; doi:10.1371/journal.pdig.0000787)
Supplement: S2 File — (ZIP) [file pdig.0000787.s002.zip › S2 Data Raw data files containing unprocessed question inputs and LLM outputs including transcripts of deliberations/STEP3_Question-3-1_Type-MC-NJ.docx]

**Facilitator** **[Round 1]:** A 12-year-old girl is brought to the emergency department by her mother because of a 1-week history of worsening swelling in her legs. The patient also noticed blood in her urine yesterday. The bleeding has not recurred. She had an upper respiratory tract infection and sore throat 1 week ago that caused her to miss several days of school. Medical history is otherwise unremarkable and she takes no routine medications. Menarche has not yet occurred. BMI is 20 kg/m². Vital signs are temperature 37.0°C (98.6°F), pulse 78/min, respirations 12/min, and blood pressure 136/84 mm Hg. Pulse oximetry on room air shows an oxygen saturation of 100%. Physical examination discloses erythema of the posterior pharynx, mild cervical lymphadenopathy, and 3+ pitting edema to both knees. Results of urinalysis are shown: Protein: 150 mg/dL Blood: Positive Leukocyte esterase: Positive Nitrite: Negative WBCs: 5−10/hpf RBCs: 10−25/hpf Casts:1−2/lpf Results of which of the following laboratory studies are most likely to be abnormal in this patient? (A) Bleeding time (B) Erythrocyte count (C) Serum concentration of C3 (D) Serum IgA concentration (E) Serum rheumatoid factor assay

**AI_C:** RESTATING THE QUESTION: Let's analyze the symptoms laid out in the question. A young girl is experiencing lower extremity edema, microscopic hematuria, and proteinuria following a recent upper respiratory tract infection. The query is asking which among the listed laboratory tests are expected to show abnormal results based on her clinical picture. PLANNING: Understanding the girl's clinical picture postulates a potential renal condition. We hypothesize on a condition known as post-streptococcal glomerulonephritis (PSGN), a common cause of acute nephritic syndrome in children. This occurs 1-2 weeks after a streptococcal infection and is characterized by hematuria, edema, and hypertension, symptoms noted in our patient. Once we suspect PSGN, we question what are the abnormalities that we might expect to see in laboratory findings? CLINICAL REASONING: PSGN triggers an immune reaction that leads to kidney damage. Part of this process involves the activation of the complement system, a group of serum proteins that aid antibodies and phagocytes in clearing pathogens. Specifically, heightened immune response could cause overuse and depletion of complement component 3 (C3). When considering this, it leads us to hypothesize that there might be a decrease in the serum concentration of C3. CONCLUDING: Following the clinical reasoning of the suspected diagnosis and understanding the pathogenesis of PSGN, we associate a decrease in C3 levels as a common lab finding in PSGN. This could help to further confirm our suspected diagnosis. RESPONSE: The laboratory study most likely to exhibit abnormal results in this patient would be the serum concentration of C3 (Choice C), which may be reduced as part of the immune complex-mediated activation of the complement pathway triggered by the post-streptococcal infection.

**AI_D:** RESTATING THE QUESTION: A 12-year-old girl exhibiting leg swelling, hematuria, and a previous recent upper respiratory tract infection presents to the emergency department. These findings along with elevated protein and blood levels in her urinalysis suggest that she may have a kidney-related issue. The question is asking which additional laboratory tests could potentially reveal abnormal results considering her symptoms and signs. The choices are: (A) Bleeding time: duration it takes for a standardized wound to stop bleeding. (B) Erythrocyte count: number of red blood cells (RBCs) in a volume of blood. (C) Serum concentration of C3: levels of complement component C3, a protein involved in the immune response. (D) Serum IgA concentration: levels of immunoglobulin A (IgA), a type of antibody. (E) Serum rheumatoid factor assay: testing for the presence of rheumatoid factor (RF), an antibody found in rheumatoid arthritis. PLANNING: The two main possibilities for this patient’s presentation are poststreptococcal glomerulonephritis (PSGN) or nephritic syndrome related to a different cause. Considering her symptoms to be primarily kidney-related is an assumption, but her physical presentation and urinalysis suggest renal involvement. The alternative assumption could be that her symptoms are the result of an unrelated systemic process, but the presence of renal casts, hematuria and proteinuria make this unlikely. CLINICAL REASONING: A more likely explanation is that the girl's recent history of upper respiratory tract infection and sore throat could have been caused by Group A Streptococcus infection, which can consequently lead to PSGN. This aligns with the assumption that her symptoms may be primarily kidney-related, and this disease often presents with periorbital and lower extremity edema, hypertension, and hematuria – all of which this patient has – as well as potentially abnormal levels of serum C3 due to the involvement of the immune system’s complement pathway in PSGN. CONCLUDING: The girl’s recent potential exposure to streptococcal infection followed by her display of the common signs of nephritic syndrome make PSGN a likely diagnosis, and this possibility would be supported by the presence of abnormal C3 serum concentration, as it is a part of the immune response against the streptococcal antigen. The other options do not seem as immediately relevant given her presentation and the pattern of her symptoms. RESPONSE: The most clinically likely abnormal test result for this patient would be the serum concentration of C3 (C).

**AI_B:** RESTATING THE QUESTION: We are being asked to identify the likely abnormal laboratory finding in a 12-year-old girl who, in the past week, has experienced worsening leg swelling and detected blood in her urine. Concurrently, she has experienced an upper respiratory infection and a sore throat. There are indications that this patient has kidney issues, as shown by the elevated levels of protein, blood, leukocyte esterase, white blood cells (WBCs), red blood cells (RBCs), and casts in her urine, a condition known as nephritic syndrome. The patient's abnormal blood pressure and increased protein levels in the urine are causes for concern. This patient's lab results may reveal that she has a variation in her: (A) Bleeding time (B) Erythrocyte (red blood cell) count (C) C3 complement protein serum concentration (D) IgA (an antibody) serum concentration (E) Rheumatoid factor presence in her serum PLANNING: This patient's symptoms—leg swelling, blood in urine, recent upper respiratory tract infection—suggest poststreptococcal glomerulonephritis, a type of nephritic syndrome. This condition often follows streptococcal infection and leads to inflammation of the glomeruli in the kidneys, causing protein and blood in urine. Corresponding laboratory findings commonly include a decrease in serum concentration of complement protein C3 due to its consumption in the immune response. CLINICAL REASONING: The patient's reported symptoms align with poststreptococcal glomerulonephritis: leg edema (due to kidney dysfunction and fluid retention), hematuria (red blood cells in the urine due to glomerular damage), and recent pharyngitis (commonly caused by streptococci). In such cases, the immune response to streptococcal infection involves the activation of the complement system, a component of the innate immunity. Specifically, the C3 component is utilized, leading to its decrease in serum. Thus, abnormality in C3 serum concentration is expected. CONCLUDING: Given the patient's symptoms, the abnormality is most likely to be found in the serum concentration of C3. The decrease in this component reflects its consumption during the immune response to the streptococcal infection, leading to poststreptococcal glomerulonephritis, a diagnosis supported by the patient's symptoms and the urinalysis results. RESPONSE: Of the given laboratory studies, the serum concentration of C3 (option C) is the most likely to be abnormal in this patient, reflecting a decrease due to its consumption in poststreptococcal glomerulonephritis.

**AI_E:** Restating the question: We are tasked with identifying which laboratory test might be abnormal in a 12-year-old girl who's experiencing leg swelling and has previously noticed blood in her urine. She has a recent history of an upper respiratory infection and sore throat. Her urinalysis indicates the presence of protein, blood, leukocyte esterase, WBCs, RBCs, and casts. She has a normal body mass index, a fever, a slightly high pulse rate, and normal oxygen saturation. Her physical exam discloses erythema of the posterior pharynx, mild cervical lymphadenopathy, and significant pitting edema up to her knees. The technical terms involved in this scenario include upper respiratory tract infection (inflammation of the airways leading to the lungs), pitting edema (a condition where pressing on the swollen area leaves an indentation), urinalysis (a broad range of tests performed on urine), leukocyte esterase (an enzyme present in certain white blood cells), nitrite (a substance usually associated with a urinary tract infection), WBCs (white blood cells, usually elevated in infections), RBCs (red blood cells, the presence of which in urine may suggest a problem with the kidneys or urinary tract), and casts (solidified protein material that form in the renal tubules and may indicate kidney damage). Planning: The symptoms might indicate a postinfectious glomerulonephritis (PIGN), which often presents in children following a Group A Streptococcus infection of the throat, manifesting as edema due to proteinuria and hematuria. This condition is typically characterized by hypoalbuminemia leading to edema, an elevation of the antistreptolysin O (ASO) titer, and reduced complement proteins. This leads us to suspect that the patient's serum concentration of C3 will likely be abnormal, rather than bleeding time, serum IgA concentration, serum rheumatoid factor assay, or erythrocyte count. Clinical Reasoning: PIGN is caused by the deposition of immune complexes that stimulate inflammation and damage the glomerular structures of the kidneys, leading to the leakage of protein and blood into the urine, as observed in this patient's urinalysis. This immune reaction often includes the activation of the complement system, and particularly the C3 protein. Hence, there is a tendency for C3 serum concentrations to fall during an active disease state as the immune system uses up the complement proteins to manage the inflammation. Therefore, in this livedo, the patient is expected to show a reduced serum C3 level. Concluding: It's essential to remember that blood tests are used to measure levels of different cells and chemicals in the blood. The blood test results help doctors to diagnose and monitor many different diseases and conditions. As for this patient, the symptoms and urine tests indicate that there might be an inflammation and damage to the kidneys. One of the proteins involved in these reactions is the C3 protein. Since it's used up in the process, it's expected to be lower in the patient's blood. Response: Given these considerations and the clinical picture of a potential post-streptococcal glomerulonephritis scenario, the most likely laboratory abnormality would be a low serum concentration of C3 (C).

**AI_A:** RESTATING THE QUESTION: This query centers around which laboratory test would likely give abnormal results for a 12-year-old girl with signs indicative of a renal condition. Following an upper respiratory tract infection, she presents with bilateral leg swelling, blood in her urine, erythema of the posterior pharynx, mild cervical lymphadenopathy, and pitting edema. Hematuria and proteinuria are evident from the urinalysis report. The patient doesn't have signs of bleeding diathesis, anemia, increased serum IgA or rheumatoid factor activity from the case details. She shows symptoms suggestive of a post-infectious glomerulonephritis related to recent sore throat and upper respiratory tract infection. PLANNING: The essential question we should be asking is which of these lab tests is likely to be abnormal in the context of post-infectious glomerulonephritis caused by, for instance, a Streptococcal infection. The tests in due consideration are: bleeding time, erythrocyte count, serum concentration of C3 complement, serum IgA concentration, and serum rheumatoid factor assay. The different ailments associated with the results of these tests are bleeding disorders, anemia, immune system disorders including autoimmune diseases and recurrent bacterial infections, IgA nephropathy/Berger’s disease, and rheumatoid arthritis respectively. The most related condition here given her clinical presentation is an immune system disorder, possibly triggering an inflammation of the glomeruli in her kidneys. The complement system, particularly C3, is directly involved in such immune reactions and is usually consumed during this process thereby reducing its serum concentration. CLINICAL REASONING: Given that the patient presented here has leg swelling (edema), and results of her urinalysis show proteinuria and hematuria (protein and blood in urine) following a throat infection, it implies an ongoing kidney issue. Proteinuria and edema hint towards a loss of protein in the urine compromising the oncotic pressure balance leading to edema. A typical case of post-infectious glomerulonephritis can present this way, particularly after streptococcal infection. The activation of the immune response due to the infection can result in glomerular damage facilitated by immune complexes deposition and complement activation. This immune response causes a decline in the complement component C3 concentration - a fundamental part of the immune system's functionality. CONCLUDING: The patient's history, symptoms, physical signs, and urinalysis results strongly suggest the possibility of post-infectious glomerulonephritis, and this condition can result from a hyperactive immune reaction to a recent infection. The immune reaction in such cases often leads to a decrease in the concentration of complement proteins, primarily C3. Therefore, we hypothesize that the serum concentration of C3 is most likely to be abnormal in this patient. RESPONSE: So, in the context of this patient's symptoms and history, it is likely that the serum concentration of C3 (Option C) might be abnormal as it might be decreased due to consumption by the immune system responding to post-infectious glomerulonephritis.

**Facilitator** The consensus statement synthesizes the responses of AI_A, AI_B, AI_C, AI_D, and AI_E as follows: The question pertains to a 12-year-old girl who presents with symptoms indicative of a renal condition, including leg swelling and blood in her urine, following an upper respiratory tract infection. The urinalysis report reveals proteinuria and hematuria. The laboratory tests under consideration are bleeding time, erythrocyte count, serum concentration of C3 complement, serum IgA concentration, and serum rheumatoid factor assay. The patient's symptoms, history, and urinalysis results suggest a diagnosis of post-infectious glomerulonephritis, likely due to a recent streptococcal infection. This condition is characterized by an immune response that can lead to kidney damage, often involving the activation of the complement system, specifically the C3 component. This immune response typically results in a decrease in serum C3 concentration due to its consumption in the immune response. Therefore, the consensus among the AI responses is that the most likely abnormal laboratory finding in this patient would be a decrease in the serum concentration of C3. This is due to its involvement and consumption in the immune response to the post-infectious glomerulonephritis, which aligns with the patient's symptoms and urinalysis results.
